# Are quantitative trait-dependent sampling designs cost-effective for analysis of rare and common variants?

**DOI:** 10.1186/1753-6561-5-S9-S111

**Published:** 2011-11-29

**Authors:** Yildiz E Yilmaz, Shelley B Bull

**Affiliations:** 1 Samuel Lunenfeld Research Institute of Mount Sinai Hospital, Prosserman Centre for Health Research, 60 Murray Street, Box 18, Toronto, ON M5T 3L9, Canada; 2 Dalla Lana School of Public Health, University of Toronto, Health Sciences Building, 155 College Street, Toronto, ON M5T 3M7, Canada

## Abstract

Use of trait-dependent sampling designs in whole-genome association studies of sequence data can reduce total sequencing costs with modest losses of statistical efficiency. In a quantitative trait (QT) analysis of data from the Genetic Analysis Workshop 17 mini-exome for unrelated individuals in the Asian subpopulation, we investigate alternative designs that sequence only 50% of the entire cohort. In addition to a simple random sampling design, we consider extreme-phenotype designs that are of increasing interest in genetic association analysis of QTs, especially in studies concerned with the detection of rare genetic variants. We also evaluate a novel sampling design in which all individuals have a nonzero probability of being selected into the sample but in which individuals with extreme phenotypes have a proportionately larger probability. We take differential sampling of individuals with informative trait values into account by inverse probability weighting using standard survey methods which thus generalizes to the source population. In replicate 1 data, we applied the designs in association analysis of Q1 with both rare and common variants in the *FLT1* gene, based on knowledge of the generating model. Using all 200 replicate data sets, we similarly analyzed Q1 and Q4 (which is known to be free of association with *FLT1*) to evaluate relative efficiency, type I error, and power. Simulation study results suggest that the QT-dependent selection designs generally yield greater than 50% relative efficiency compared to using the entire cohort, implying cost-effectiveness of 50% sample selection and worthwhile reduction of sequencing costs.

## Background

We assume an existing cohort of unrelated individuals, well phenotyped for a quantitative trait (QT) that is approximately normally distributed. At present, the cost of whole-genome sequencing of an entire cohort to test for association remains prohibitive, and selection of a subset of informative individuals for sequencing is a reasonable strategy to reduce costs. In the absence of relevant genetic information, such as family history or single-nucleotide polymorphism (SNP) association identified through genome-wide association studies, one might hypothesize that individuals at the extremes of the QT distribution would be more informative for the detection of association with sequence variants. For example, individuals with high QT values are more likely to carry high-risk variants, whereas those with low values do not. In contrast, taking a simple random sample from the cohort will tend to select individuals from the central part of the trait distribution and can fail to sample individuals with low-frequency variants. Because ascertainment by the phenotype needs to be taken into account, simple selection from the tails of the trait distribution complicates inference. The resulting trait distribution is bimodal, violating the usual linear regression assumptions and can produce distorted effect estimates and type I errors greater or less than nominal. In addition, the resulting sample is not representative of the original cohort, making it more difficult to design validation studies in nonselected populations. Finally, if both extremely high and extremely low QT values are “pathological” then excluding “normal” individuals would reduce the ability to detect protective variants associated with favorable QT values. As an alternative, we propose a sampling design in which all individuals have a nonzero probability of being selected into the sample but in which individuals with extreme phenotypes have a proportionately larger probability of being selected. We account for differential sampling in the association analysis by inverse probability weighting (IPW) with standard survey methods for variance estimation and hypothesis testing. The purpose of our analyses of the Genetic Analysis Workshop 17 (GAW17) mini-exome data is to compare alternative sampling strategies with respect to relative efficiency. We also examine type I error and power of hypothesis testing.

## Methods

### Sampling designs

We consider the analysis of sequencing data for the entire cohort as the ideal against which to compare sampling designs in which only 50% of the cohort is sequenced. Four alternative sampling designs are summarized in Table 1. Extreme-phenotype alternative designs 3 and 4 are systematic and do not actually involve any random selection; they differ only in whether individuals with “normal” values are chosen for sequencing. Design 2 involves simple random sampling (SRS) and thus is expected to be unbiased compared to the entire cohort, but it has reduced power and is subject to sampling variation. Under design 5, which is similarly subject to sampling variation, we specify the sampling probability for each individual according to the value of their quantitative trait *Y_i_*. For individual *i*, we use the distance from the median of the trait distribution (*Y*_med_) in the original cohort:(1)

In particular, we specify the IPW weight as:(2)

where the sampling probabilities under design 5 are:(3)

or:(4)

which we refer to as 5.1 and 5.2, respectively. These probabilities are scaled by the maximum distance from the median, with:(5)

where *Y*_max_ and *Y*_min_ are the maximum and minimum of the *Y_i_*, respectively. In design 5.2 extreme observations are selected more frequently.

**Table 1:** Sampling designs and analytical methods for QT association analysis

Sampling designs	Method of analysis		Software
		
	Common variant	Rare variant score	
1. Entire cohort (100%) 2. 50% simple random sample 3. All observations in each of 25% tails of the QT distribution 4. All observations in each of 20% tails and central 10% of QT distribution 5. 50% sample by distance from the median of the QT distribution	a. Linear regression of QT on genotype b. Logistic regression of genotype with QT as covariate	c. Linear regression of QT on rare allele count d. Poisson regression of rare allele count with QT as covariate	Designs 1–5: generalized linear regression (glm) function in R for fitting all models Design 5 for methods a and c: svyglm function in R with inverse probability weights

### Statistical models and hypothesis testing

For common variants, it is feasible to analyze one SNP at a time. For rare variants, however, methods that accumulate multiple SNP genotypes across a region are necessary to improve the power to detect association. We calculate a rare variant score, defined as the total count of rare alleles within a gene [1,2]. Association analyses of a QT under designs 1 and 2 are conducted using standard linear regression methods with the QT as the dependent variable. To account for unequal sampling probabilities under design 5, we apply IPW in the linear regression model parameter estimation and use the R function svyglm from the survey package to estimate appropriate variances and construct a test statistic [3]. However, because we select observations according to values of QT, in designs 3, 4, and 5 the validity of standard linear regression methods is questionable. We hypothesize that reversing the direction of the regression is a potentially more robust alternative. As described in other contexts, reversing the regression has the advantage of conditioning out the phenotype. When analyzing a common variant, we model the genotype(s) conditional on the QT value using logistic regression, and when analyzing a rare variant score, we model the count conditional on the QT value using Poisson regression.

### Application to GAW17 data

We analyze quantitative traits Q1 and Q4 in replicates 1 to 200 of the GAW17 simulated unrelated mini-exome data [4]. With knowledge of the generating model for Q1, we chose the SNPs in the *FLT1* gene to compare methods under a true alternative hypothesis of genetic association and use analysis of Q4 for comparisons under the null hypothesis. To reduce heterogeneity and potential for population stratification bias, we chose the Asian subpopulations for study: Chinese + Japanese (*n* = 321).

We excluded SNPs in the *FLT1* gene that were monomorphic within the Asian subpopulation. Of the 20 remaining SNPs, 18 were classified as rare (all minor allele frequencies [MAFs] were less than 3.0%); but according to the generating model, only 6 of these had functional variants. We calculated a rare variant score (the count of rare alleles) from the 18 rare SNPs. The total count of rare alleles per person ranged from 0 to 3 (with corresponding frequencies of 252, 54, 11, and 4). One SNP with a common functional variant, C13S523, was analyzed separately (MAF = 8.72% with no rare homozygotes).

QT-dependent sampling and QT analysis for Q1 and for Q4 were based on residuals from a linear regression on Age, Sex and Smoking status. We conducted an association analysis under each of the five sampling designs listed in Table 1 in all 200 replicates. Results are reported for replicate 1 alone to illustrate application of the methods in a single data set. We summarized distributions of regression coefficients (means and variances), standard error estimates (means), and test statistics (type I error and power) across replicates.

## Results

### Analysis of Q1 with *FLT1* SNPs in replicate 1

Association of Q1 with *FLT1* was detected at near genome-wide significance levels for both the common SNP and the rare variant score (Table 2) using the entire Asian subpopulation. The logistic regression test statistics are nearly always smaller than the linear regression test statistics, as expected, because logistic regression is less efficient for a normally distributed trait. Under linear regression analysis, association signals for all of the 50% designs were attenuated, with the most attenuation for designs 2 and 5 and the least attenuation for extreme-phenotype designs 3 and 4. However, the regression coefficients for designs 3 and 4 appear to be inflated. Under logistic regression analysis, design 5 had the least signal attenuation, and under Poisson regression it had less signal attenuation when extreme phenotypes were selected more frequently, as in design 5.2.

**Table 2:** Results of regression analysis of Q1 with the *FLT1* gene in replicate 1

Sampling design	Linear regression				Poisson regression			
	
	Coefficient	SE	Test statistic	*p*-value	Coefficient	SE	Test statistic	*p*-value
Rare variant score								
1	0.44	0.08	5.18	4 × 10^−7^	0.60	0.11	5.59	2 × 10^−8^
2	0.43	0.11	3.81	2 × 10^−4^	0.55	0.13	4.14	3 × 10^−5^
3	0.71	0.14	4.94	2 × 10^−6^	0.68	0.13	5.26	1 × 10^−7^
4	0.80	0.14	5.59	1 × 10^−7^	0.79	0.14	5.81	6 × 10^−9^
5.1	0.30	0.11	2.82	5 × 10^−3^	0.61	0.11	5.37	8 × 10^−8^
5.2	0.15	0.10	1.48	0.14	0.63	0.11	5.58	2 × 10^−8^
Common variant C13S523								
1	1.00	0.12	8.11	1 × 10^−14^	1.39	0.21	6.51	8 × 10^−11^
2	0.96	0.18	5.24	5 × 10^−7^	1.40	0.32	4.36	1 × 10^−5^
3	1.67	0.21	7.95	3 × 10^−13^	1.66	0.34	4.88	1 × 10^−6^
4	1.71	0.21	8.17	9 × 10^−14^	1.86	0.37	5.01	5 × 10^−7^
5.1	0.90	0.17	5.45	2 × 10^−7^	1.39	0.27	5.18	2 × 10^−7^
5.2	0.26	0.22	1.16	0.25	1.39	0.26	5.44	5 × 10^−8^

*P*-values are based on the asymptotic distribution of the test statistic.

### Evaluation of test statistics in replicates 1–200

We evaluated type I error at the nominal 5% level by means of association analysis of Q4 with the *FLT1* rare variant score and common causal variant. With 200 replicates, the 95% confidence interval is roughly 0.05 ± 0.03. For the rare variant score, the linear regression type I error tended to be less than 5% (Table 3), except for design 5.2, in which the empirical standard deviation was larger than the mean standard error (SE), yielding an elevated type I error. In Poisson regression, type I error was somewhat elevated, with the generalized linear model SE estimates too small on average. For the common variant (data not shown), type I error was within 5% for both linear and logistic regressions, except for design 5.2. In this case, in which the sampling probabilities had a wide range, the survey variance estimation method applied in the linear regression tended to underestimate the empirical variance, as also observed in the rare variant analysis. For logistic regression, however, design 5.2 was equivalent to designs 3 and 4.

**Table 3:** Simulation results for analysis of Q4 with the *FLT1* rare variant score, replicates 1–200

Sampling design	Linear regression				Poisson regression			
	
	Mean coefficient	Empirical SD	Mean SE	Type I error	Mean coefficient	Empirical SD	Mean SE	Type I error
	
1	−0.0014	0.042	0.044	0.040	−0.0089	0.254	0.233	0.075
2	−0.0044	0.060	0.062	0.040	−0.0280	0.364	0.333	0.065
3	−0.0025	0.083	0.086	0.040	−0.0076	0.270	0.246	0.060
4	−0.0031	0.079	0.083	0.030	−0.0127	0.274	0.252	0.050
5.1	−0.0007	0.045	0.046	0.045	−0.0025	0.304	0.281	0.070
5.2	−0.0004	0.051	0.046	0.105	−0.0142	0.274	0.251	0.070

SD, standard deviation. SE, standard error.

**Figure 1:**
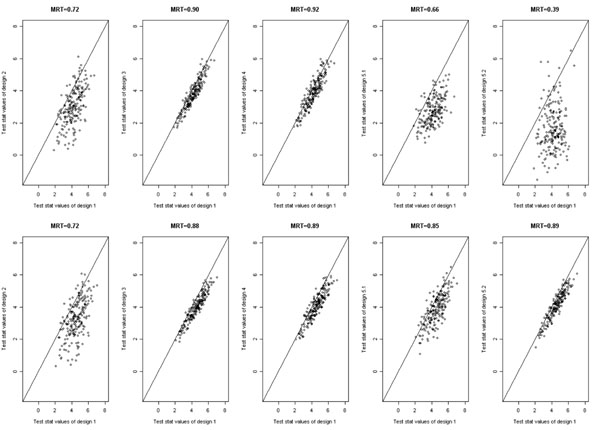
**Linear (upper panels) and Poisson (lower panels) regression test statistics in Q1 rare variant analysis. **MRT, mean ratio of test statistics.

To graphically convey power differences, we constructed pairwise comparison plots of design 1 test statistics for Q1 and the alternative designs in the 200 replicates. We also calculated mean values of the ratio of the design 1 test statistic over each of the test statistics for the alternative designs that we referred to as the mean ratio of test statistics (MRT). For the rare variant score (Figure 1), loss of power under design 2 is readily apparent in both linear and Poisson regressions. The scatter observed in designs 2, 5.1, and 5.2 can be explained partly by the additional variation associated with the random sampling component. The test statistics for extreme-phenotype designs 3 and 4 were strongly correlated with those for design 1, with high MRT values. Under Poisson regression, MRT values for designs 5.1, and 5.2 were similarly high. For the common variant (data not shown), discrepancies with design 1 test statistics were generally similar to those for the rare variant analysis, with equivalent performance for all designs under logistic regression but substantial loss of power.

### Evaluation of estimation efficiency using replicates 1–200

**Figure 2:**
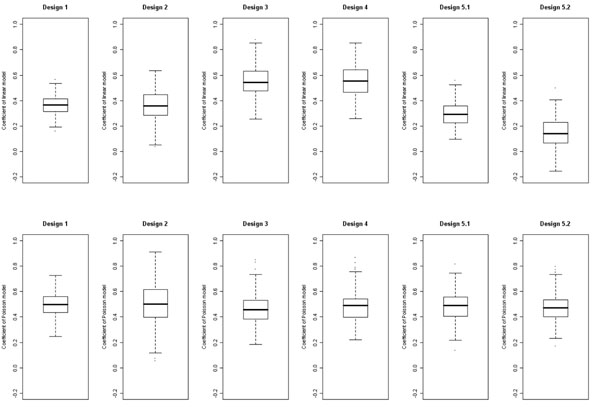
Linear (upper panels) and Poisson (lower panels) regression coefficients in Q1 rare variant analysis

Under the null analysis of the rare variant score for Q4, all estimates were close to being unbiased (Table 3). For Q1, however, when extreme-phenotype values were oversampled and this was ignored (under designs 3 and 4), the mean coefficients in the linear regression were 50% larger than those under design 1 (Figure 2). In contrast, the mean coefficients for Poisson regression were similar under designs 1, 3 and 4. Notably, when we incorporated the sampling probability of each individual according to the value of their QT, as in sampling design 5, the mean coefficient was attenuated in the linear regression but close in value to that for the entire sample in Poisson regression. Similar findings were obtained for common variant linear and logistic regressions (data not shown).

To compare designs in terms of precision of estimation, we evaluated relative efficiency (RE), defined as 100 times the ratio of the empirical variance of design 1 to that of the alternative design (Table 4). This measure does not depend on SE estimation but may be affected by differences in the expected value of the regression coefficient, so we considered the null case of Q4, in which estimates were approximately unbiased, and the alternative case of Q1. In comparison to analysis of the entire cohort, we observed only 40–50% RE under SRS design 2. Under extreme-phenotype designs 3 and 4, RE was low for linear regression but high for logistic and Poisson regression. In contrast, linear regression REs for Q1 and Q4 were highest for sampling design 5.1, with an RE of 88% for Q4. However, when extreme observations were selected with high probability, as in design 5.2, RE was high for logistic and Poisson regression and comparable to that of designs 3 and 4.

**Table 4:** Relative efficiencies of regression coefficients for analysis of Q1 and Q4 with *FLT1*, replicates 1–200

Sampling design	Rare variant score				Common variant			
	
	Linear regression		Logistic regression		Linear regression		Poisson regression	
	Q4	Q1	Q4	Q1	Q4	Q1	Q4	Q1
2	50	41	49	43	46	46	45	45
3	26	36	89	70	26	55	87	50
4	29	34	86	68	26	50	78	53
5.1	88	62	70	53	88	57	72	46
5.2	70	44	86	76	56	31	84	52

## Discussion

As expected, use of a 50% SRS design is not a cost-effective approach for reducing sequencing costs: The empirical relative efficiency of estimation was less than 50% in the GAW17 simulations we examined. However, designs 3–5, in which RE generally exceeded 50% when conditioning on the QT and could be substantially higher, appeared to offer a net benefit. The more extreme IPW sampling design 5.2, which is similar to designs 3 and 4, behaves similarly, at least for the regression analyses that condition on the phenotype. However, the simple IPW linear regression analysis that we applied here may be less than optimal in finite samples under the alternative hypothesis. Although RE results for design 5 are encouraging, for hypothesis testing the source of parameter underestimation under the alternative hypothesis needs to be addressed and SE estimation improved. Evaluations reported here are limited, and it is quite possible that sampling and/or weighting schemes could be improved.

In our extreme-phenotype implementation of designs 3 and 4, we used the observed QT values, in contrast to more common case-control approaches. Because the linear regression assumption of normal residuals is grossly violated, the observed exaggeration of regression coefficients is not surprising. The lack of elevated type I error may be ascribed to the use of symmetric tail selection, but for rare variant analysis there is a clear loss of efficiency. In the GAW17 simulation, in which rare and common variant effects were generated under an additive model, differences between designs 3 and 4 were trivial. In other settings, design 4 may be more robust.

Although it has not been commonly applied, Poisson regression of allele counts in the rare variant score appears to be useful as a general approach for single-gene analysis in many designs, provided that the tendency toward elevated type I error is resolved. For design 5, Poisson regression avoids the complications of weighting, because the model conditions on the QT value.

## Conclusions

There is established literature on optimal design for continuous outcomes in experimental settings and survey samples, and sampling has been well exploited in the context of time-to-event outcomes in epidemiological designs, such as the case-cohort study [5]. In comparison to using the entire cohort, extreme sampling incurs some loss of efficiency and power to detect association, but it is far more efficient than simple random sampling of the same number of individuals. Although our comparisons in the GAW17 data do not conform to a wholly realistic setting in terms of sample size and volume of sequencing data, they do suggest that QT-dependent sampling can be quite effective in reducing sequencing costs with developing prospects for whole-genome sequencing.

## Competing interests

The authors declare that there are no competing interests.

## Authors’ contributions

YEY and SBB conceived of the study. YEY carried out the analyses and wrote the first draft of the manuscript. Both authors revised and approved the final manuscript.
